# TP53I11 Functions Downstream of Multiple MicroRNAs to Increase ER Calcium Levels and Inhibits Cancer Cell Proliferation

**DOI:** 10.3390/ijms26010031

**Published:** 2024-12-24

**Authors:** Yiping Wang, Shuai Zhang, Jie Bing, Wanjie Li, Lin Sun, Youjun Wang

**Affiliations:** 1 Beijing Key Laboratory of Gene Resource and Molecular Development, College of Life Sciences, Beijing Normal University, Beijing 100875, Chinabingjie@bnu.edu.cn (J.B.); lwj@bnu.edu.cn (W.L.); sunlin@bnu.edu.cn (L.S.); 2 Key Laboratory of Cell Proliferation and Regulation Biology, Ministry of Education, College of Life Sciences, Beijing Normal University, Beijing 100875, China

**Keywords:** endoplasmic reticulum, Ca^2+^ homeostasis, microRNA, doxorubicin, TP53I11

## Abstract

Cells meticulously regulate free calcium ion (Ca^2+^) concentrations, with the endoplasmic reticulum (ER) being crucial for Ca^2+^ homeostasis. Disruptions in ER Ca^2+^ balance can contribute to various diseases, including cancer. Although considerable research has focused on the direct mechanisms of ER Ca^2+^ regulation, the role of microRNAs (miRNAs) in this process remains underexplored. Mainly using data from a CRISPR-based genomic screening previously conducted in our laboratory, we identified 33 candidate miRNAs that may regulate ER Ca^2+^ levels. From these, 10 miRNAs were found to significantly lower basal ER Ca^2+^ levels. RNA sequencing analysis indicated that these miRNAs downregulate the tumor suppressor tumor protein p53 (TP53)-inducible protein 11 gene (*TP53I11*), which is a key regulator of ER Ca^2+^ levels. Functional assays confirmed that *TP53I11* influences ER Ca^2+^ levels and affects cancer cell proliferation. Additionally, the chemotherapeutic agent doxorubicin (DOX) was shown to upregulate *TP53I11* and enhance ER Ca^2+^ accumulation. These findings elucidate the central role of TP53I11 in miRNA-mediated regulation of ER Ca^2+^ homeostasis and suggest potential therapeutic strategies targeting ER Ca^2+^ upregulation for cancer intervention.

## 1. Introduction

Under physiological conditions, cells precisely regulate the concentration of free calcium ions (Ca^2+^) across different subcellular compartments [1,2]. The endoplasmic reticulum (ER) is particularly crucial in maintaining Ca^2+^ homeostasis, serving as a central reservoir that generates precise Ca^2+^ signals essential for cellular functions [3,4,5]. Disruptions in ER Ca^2+^ homeostasis can profoundly affect cellular signaling pathways and contribute to a range of diseases, including cancer, kidney disorders, and neurodegenerative conditions [6,7,8].

ER Ca^2+^ homeostasis is maintained through both direct and indirect mechanisms, which regulate the expression and activity of the protein machinery of the Ca^2+^ signaling system. The ER Ca^2+^ level is directly determined by the balance between the sarcoplasmic/ER Ca^2+^ ATPase (SERCA) pump, which actively transports Ca^2+^ from the cytoplasm into the ER [9], and the ER channels that mediate Ca^2+^ releases into the cytosol. These channels include inositol 1,4,5-trisphosphate receptors (IP_3_Rs), ryanodine receptors (RYRs), and passive leakage channels [10,11,12,13,14]. When the ER Ca^2+^ pool is excessively high, cells may also release Ca^2+^ through the ER Ca^2+^ load-activated Ca^2+^ (CLAC) channel [15,16]. Additionally, ER Ca^2+^ homeostasis is indirectly influenced by fluctuations in cytoplasmic Ca^2+^ levels. Plasma membrane Ca^2+^ channels and Ca^2+^ release-activated Ca^2+^ (CRAC) channels facilitate Ca^2+^ influx, while plasma membrane Ca^2+^-ATPase (PMCA) and Na^+^/Ca^2+^ exchangers (NCX) are responsible for exporting Ca^2+^ from the cytoplasm [7,17,18,19,20]. Furthermore, organelles such as mitochondria and the Golgi apparatus contribute to ER Ca^2+^ regulation by modulating Ca^2+^ transport and storage.

While substantial research has been focused on the regulation of ER Ca^2+^ homeostasis [21,22,23,24,25,26,27,28,29,30], the role of microRNAs (miRNAs) has received less attention. miRNAs are noncoding RNAs, approximately 21–23 nucleotides in length, which regulate gene expression by binding to target mRNAs and inhibiting their translation [31,32,33,34]. Several miRNAs have been identified as key regulators of ER Ca^2+^ homeostasis, affecting Ca^2+^ handling proteins such as SERCA, IP_3_Rs, RYR, stromal interaction molecules (STIMs), Orai, NCX, and the mitochondrial Ca^2+^ uniporter (MCU) [35,36,37,38,39,40,41]. However, research into miRNA-mediated regulation of ER Ca^2+^ homeostasis remains fragmented and lacks systematic exploration, partly due to the limited availability of sensitive sensors for ER Ca^2+^ levels.

The aim of this study is to systematically investigate the role of miRNAs in the regulation of ER Ca^2+^ homeostasis. By combining the use of our newly developed miGer and TuNer-s, highly sensitive ratiometric ER Ca^2+^ indicators [42,43], with a CRISPR-based genomic screening, we recently obtained a pool of 33 candidate miRNAs that may affect ER Ca^2+^ levels [44]. From this library, we pinpointed 10 miRNAs that significantly lower basal ER Ca^2+^ levels. RNAseq results showed that the tumor suppressor tumor protein p53 (TP53)-inducible protein 11 gene (*TP53I11*) is downregulated by all 10 of these miRNAs. Furthermore, we discovered that chemotherapeutic agent doxorubicin (DOX) can upregulate *TP53I11*, resulting in elevated ER Ca^2+^ levels in cancer cells. From this small pool of the library, newly identified by us, we pinpointed 10 miRNAs that significantly lower ER Ca^2+^ levels. These results not only enhance our understanding of the epigenetic regulation of ER Ca^2+^ homeostasis but also reveal its implications for related diseases, offering potential new avenues for therapeutic intervention.

## 2. Results

### 2.1. Functional Screening Led to the Identification of Ten miRNAs That Lower ER Ca^2+^ Levels

**Figure 1:**
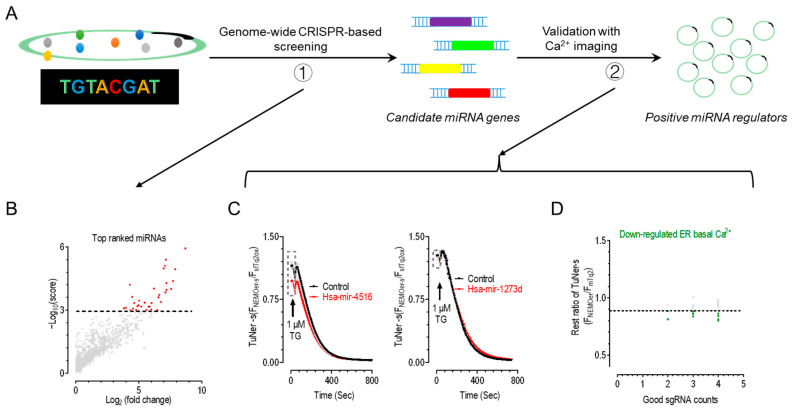
Identification of ER-Ca^2+^-lowering miRNAs from a pool of candidate ER Ca^2+^ homeostasis-regulating miRNAs. (**A**) Schematic diagram illustrating the workflow of genomic screening and imaging validation to identify ER-Ca^2+^-level-altering miRNAs from candidate library of ER Ca^2+^ homeostasis-regulating. The colored dots on the left represent single gene-edited cells. (**B**) Enriched miRNAs identified from CRISPR/cas9-edited cells with increased ER Ca^2+^ levels. The top 1% ranked genes of miRNAs determined with robust ranking aggregation (RRA) algorithm are shown in red. The dotted line represents the boundary dividing the significant and non-significant areas. (**C**,**D**) Ca^2+^ imaging assay used to identify ER-Ca^2+^-altering miRNAs from top-ranked candidates in HEK293 cells stably expressing an ER Ca2+ sensor TuNer-s (TuNer-s cells). ER Ca^2+^ levels were indicated by the ratio of TuNer-s (FNEMOer-s/FsfTq2ox). Following the acquisition of baseline, 1 μM Thapsigargin (TG) was added (indicated by arrows) to ensure full depletion of ER Ca^2+^ store. TuNer-s ratios were compared between cells exhibiting mScarlet fluorescence (the experimental group) and cells lacking mScarlet fluorescence (the internal control) within the same view field. Unless specified, all imaging experiments in this work were performed and presented the same way. Typical traces of a positive (left) and a negative (right) hit (**C**). Data are shown as mean ± SEM. Statistics showing the effects of overexpressing miRNAs on basal ER Ca^2+^ levels in TuNer-s cells. TuNer-s ratios of experimental groups were normalized to their corresponding internal controls. Green dots indicating validated miRNA genes based on the Ca^2+^ imaging described in. Conversely, gray dots denoted genes that did not exhibit statistically significant effects (**D**). Data are shown as the average of at least three independent replicates, the dotted line represents the boundary dividing the significant and non-significant areas.

**Figure 2:**
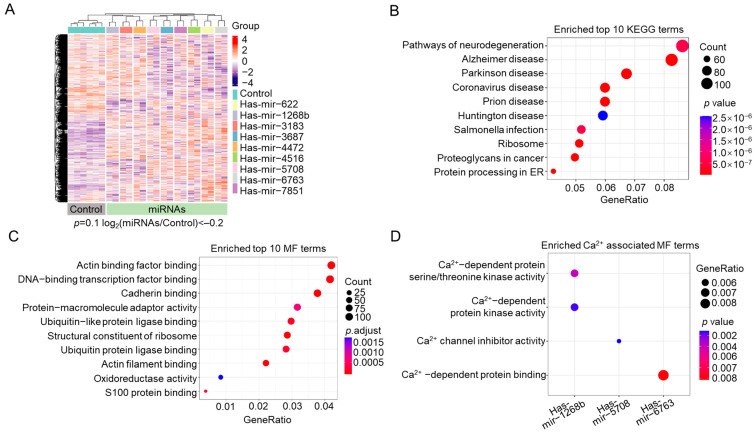
MF and KEGG analysis of differentially expressed genes (DEGs) of cells transiently overexpressing identified ER-Ca^2+^-lowering miRNAs. HEK293 cells transiently expressing either individual ER-Ca^2+^-lowering miRNAs or an empty vector underwent RNA-seq. The corresponding DEGs were subsequently analyzed for GO and KEGG pathway enrichments. (**A**) Heatmap of the differential gene expression profiles from cluster analysis of RNA-seq results at the indicated *p* with a log_2_(miRNAs/Control) < –0.2. Red and blue colors represent high and low DEGs, respectively. (**B**–**D**) KEGG pathway and MF enrichment analysis for downregulated DEGs from HEK293 cells expressing miRNAs. Dot plots showing the KEGG pathways (**B**), the top 10 (**C**), and Ca^2+^-associated MF terms (**D**).

We followed the workflow outlined in Figure 1A to systematically identify and validate miRNAs that regulate ER Ca^2+^ homeostasis. Using cells stably expressing the ratiometric Ca^2+^ indicator miGer, along with genomic-scale gene-editing techniques and high-throughput sequencing approaches, we conducted a genome-wide screen to uncover regulators of ER Ca^2+^ levels (Figure 1(A-1)) [44]. This screen identified 2208 genes associated with elevated ER Ca^2+^ levels, including 258 miRNA genes (Figure 1(A-2)). These genes were ranked using Robust Ranking Aggregation (RRA), leading to the selection of 33 candidate miRNAs from the top 10% for further analysis (Figure 1B).

We hypothesized that strong hits among these 33 candidates, which have more pronounced effects on ER Ca^2+^ levels, would exhibit opposing effects when subjected to gene knockout versus gene overexpression. Specifically, since the knockout of these 33 candidate miRNAs increased ER Ca^2+^ concentration, we anticipated that overexpression of the most impactful miRNAs would significantly reduce basal ER Ca^2+^ levels.

To identify these key miRNAs, we conducted Ca^2+^ imaging experiments using HEK TuNer-s cells, which stably express the TuNer-s Ca^2+^ indicator. TuNer-s comprises NEMOer-s, a Ca^2+^ sensor, and sfTq2^ox^, an expression reporter. ER basal Ca^2+^ levels were measured by the fluorescence ratio of these two TuNer-s components (F_NEMOer-s_/F_sfTq2ox_). This tool offers high sensitivity for detecting ER Ca^2+^ fluctuations that might be missed by conventional indicators [43]. Cells were transfected with a plasmid carrying DNA sequences for one pre-miRNA and a red fluorescent protein mScarlet, which serves as the expression marker. To assess the effects of overexpressed miRNAs, we compared the basal TuNer-s ratios from mScarlet-positive (miRNA-expressing) and mScarlet-negative (internal control) cells from the same field of view (Figure 1C, dashed boxes). To eliminate artifacts, we used 1 μM of the ER Ca^2+^ pump inhibitor, thapsigargin (TG), to fully deplete the ER Ca^2+^ store, achieving the maximal decrease in TuNer-s signals. Only cells from both groups with similar minimal TuNer-s ratio in TG were analyzed.

The results showed that 48 h post-transfection, hsa-mir-4516 significantly reduced the TuNer-s ratio, confirming its ability to reduce ER Ca^2+^ levels (Figure 1C, left). In contrast, hsa-mir-1273d showed no discernible effect on ER basal Ca^2+^ levels, as evidenced by the unchanged TuNer-s ratios (Figure 1C, right). Overall, from the 33 miRNAs tested, 10 were found to significantly reduce TuNer-s ratios, demonstrating their efficacy in decreasing basal ER Ca^2+^ levels (Figure 1D).

While the remaining 23 miRNAs did not significantly alter basal ER Ca^2+^ levels upon overexpression, it is plausible that their knockdown or knockout could still elevate basal ER Ca^2+^ concentrations. Future studies are warranted to explore this possibility. For the current investigation, we focused on the 10 miRNAs that were confirmed to effectively decrease ER basal Ca^2+^ levels when overexpressed.

### 2.2. Comprehensive RNA-Seq Analysis Reveals Target Genes and Pathways Associated with ER- Ca^2+^-Lowering miRNAs

Considering the well-known miRNA-mediated co-regulation of shared targets [45,46,47,48], we employed RNA sequencing (RNA-seq) to identify and analyze common target genes modulated by 10 ER-Ca^2+^-lowering miRNAs. RNA-seq was performed on cells expressing these miRNAs or control plasmids. Pearson correlation analysis revealed that the correlations within each group were strong, while the correlations between groups were weak (Figure S1A). This reflects a high degree of biological repeatability between sequencing samples, underscoring the consistency and reliability of the experimental data. Clustering according to Pearson correlation in R excluded miRNA groups that clustered with control cells (Figure S1B), likely due to inefficient expression and minimal effects on target gene mRNA levels. Consequently, the hsa-mir-4296 group was excluded from further analyses.

miRNAs mainly affect gene expression by binding to the 3′ untranslated region (UTR) of target mRNAs, causing mRNA cleavage and downregulation, and can also act indirectly through gene regulators. We hypothesize that downregulated differentially expressed genes (DEGs) are primarily direct miRNA targets, while upregulated DEGs are likely regulated indirectly. We conducted a differential expression analysis to categorize DEGs as downregulated (log_2_(fold change (FC)) < −0.2) [49] or upregulated (log_2_(FC) > 1). The results clearly demonstrate the presence of DEGs co-regulated by all nine miRNAs (Figure 2A).

To investigate the shared features of these miRNAs, we next conducted GO and KEGG pathway enrichment analysis of the DEGs associated with miRNA overexpression. Analysis of downregulated DEGs revealed that the top 10 most significantly enriched KEGG pathways are associated with neural or neurodegenerative diseases (hsa05016, hsa05020, hsa05022, hsa05010, and hsa05012), infection diseases and cancer (hsa05171 and hsa05205), and protein synthesis and processing (hsa04141 and hsa03010) (Figure 2B). These results highlight significant associations between these ER-Ca^2+^-lowering miRNAs and various neurodegenerative diseases, including Alzheimer’s, Parkinson’s, and Huntington’s disease.

GO analysis links DEGs to molecular functions (MF), biological processes (BP), and cellular components (CC). The top 10 enriched GO terms for the downregulated DEGs highlighted that the enriched genes are intricately involved in the regulation of protein expression. This includes modulation of gene expression through transcription factors, protein synthesis via ribosome activity and translation, and protein degradation mediated by ubiquitination. Specifically, the MF enrichment includes terms such as transcription factor binding (GO: 0140297), ubiquitin-like protein ligase binding (GO: 0044389), ubiquitin protein ligase binding (GO: 0031625), and structural constituent of ribosome (GO: 0003735) (Figure 2C). In the BP category, one notable term is related to cytoplasmic translation (Figure S2A). For CC, 7 out of 10 enriched terms are related to ribosomal components (Figure S2B).

Given the central role of ribosomes in intracellular protein synthesis, the enrichment of ribosomal subunits highlights their critical roles in protein synthesis efficiency, quality control, and potentially specific post-translational modifications. Notably, ribosomal proteins such as the ribosomal protein L18 gene (*RPL18*) and ribosomal protein S19 gene (*RPS19*) are recurrently found among these enriched terms. Additionally, 41 ribosomal protein genes were enriched across MF, BP, and CC terms. These findings suggest that alterations in these functions may impact target protein expression, indicating that these miRNAs might exert indirect regulatory effects on protein levels beyond their established roles in modulating mRNA stability.

The top enriched GO terms for downregulated DEGs also encompass proteins related to intra- or extracellular matrix functions. This includes MF terms associated with binding to actin, actin filament, and cadherin (Figure 2C); a BP term related to actin filament organization (Figure S2A); and CC terms such as the cell–substrate junction and focal adhesion (Figure S2B). Furthermore, 14 cytoskeleton and motility-related proteins, such as the filamin A gene (*FLNA*), and seven signal transduction and cell–cell interaction genes were enriched across MF, BP, and CC terms. These results suggest that the cytoskeleton and extracellular matrix might play a role in maintaining ER Ca^2+^ homeostasis.

Surprisingly, despite all tested miRNAs leading to a reduction in ER Ca^2+^ levels, the most significantly enriched MF terms did not prominently include those explicitly associated with Ca^2+^. The sole top-enriched MF term connected to Ca^2+^ homeostasis was “S100 protein binding” (GO: 0044548) (Figure 2C). Several members of the S100 protein family are well known for their roles in Ca^2+^ binding and Ca^2+^-dependent cellular processes [50]. Notably, among the 10 enriched proteins associated with S100 protein binding, 6 are critical for Ca^2+^ homeostasis. This includes three S100A homologs and two annexin A homologs that bind Ca^2+^, and an ER Ca^2+^ pump, sarcoplasmic/ER Ca^2+^ ATPase 2 gene (*SERCA2*). This suggests that pathways involving S100 proteins play a significant role in the reduction in ER Ca^2+^ levels by these miRNAs and that these miRNAs may not target a single known key regulator of ER Ca^2+^ homeostasis.

Furthermore, we analyzed Ca^2+^-related MF terms enriched in the downregulated DEGs associated with each miRNA. We found that only a few MF terms related to Ca^2+^ channels and Ca^2+^-dependent activities were enriched in just three miRNAs (Figure 2D). This observation further supports the idea that there is no common, direct target known to modulate ER Ca^2+^ levels among these miRNAs.

Subsequently, we conducted GO analysis of the upregulated DEGs to investigate whether these miRNAs might share any indirect targets known to modulate ER Ca^2+^ levels. Similarly, no prominent GO terms related to Ca^2+^ were enriched, even though a few Ca^2+^-related genes, such as Ca^2+^ voltage-gated channel subunit alpha1 A gene (*CACNA1A*), ryanodine receptor 1 gene (*RYR1*), transient receptor potential cation channel subfamily C member 4 gene (*TRPC4*), and cation channel of sperm 3 gene (*CATSPER3*), were among the most enriched terms associated with channel or transport activity (Figure S3A). Additionally, a few MF terms related to Ca^2+^ channels and transporters were enriched in only six miRNAs (Figure S3B). Collectively, these results suggest that these miRNAs may not indirectly affect a single, well-characterized regulator of ER Ca^2+^ homeostasis.

### 2.3. Identification and Validation of TP53I11 as a Common Target of ER-Ca^2+^-Lowering miRNAs

**Figure 3:**
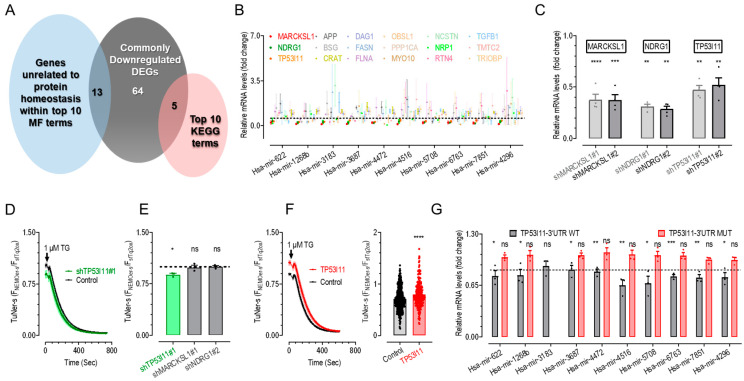
Identification of *TP53I11* as an ER Ca^2+^-increasing regulator from commonly downregulated DEGs by all 10 ER-Ca^2+^-lowering miRNAs. (**A**) Schematic illustration of strategies used to select top candidate genes from 82 commonly downregulated DEGs identified in cells expressing each of the nine tested miRNAs (grey): enriched MF terms either associated with ions or genes unrelated to protein homeostasis within top 10 terms (blue) and genes from top 10 enriched KEGG pathways (pink). The identified 18 DEGs were selected for further validation via RT-qPCR. (**B**) Scatter plot of RT-qPCR results showing the relative mRNA level of 18 candidates in HEK293 cells transiently expressing each of the ER-Ca^2+^-lowering miRNAs. The mRNA levels were normalized to GAPDH transcription, then the resulting relative mRNA levels of the candidates were further normalized against their corresponding mRNA levels in cells expressing an empty vector. The dotted line represents the boundary dividing the significant and non-significant areas. (**C**) Statistics of RT-qPCR results showing the relative mRNA levels of *MARCKSL1*, *NDRG1,* or *TP53I11* in HEK293 cells transiently expressing each of two corresponding shRNAs. The mRNA levels were normalized to GAPDH transcription, then the resulting relative mRNA levels of the candidates were further normalized against their corresponding mRNA levels in cells expressing a control shRNA (****, *p* < 0.0001; ***, *p* < 0.001; **, *p* < 0.01, unpaired Student’s *t*-test, error bars denote SEM, n = 3). (**D**,**E**) Basal ER Ca^2+^ levels in TuNer-s cells transiently expressing shTP53I11#1, shMARCKSL1#1, shNDRG1#2, or a blank vector. Typical traces of TuNer-s cells overexpressing shTP53I11#1 or a blank vector (**D**). Statistics. Basal ER Ca^2+^ levels indicated by TuNer-s ratios were normalized against corresponding blank controls. After the recording of baseline, 1 μM TG was applied to deplete ER Ca^2+^ store. The dotted line represents the boundary dividing the significant and non-significant areas (*, *p* < 0.05; ns, *p* > 0.05, unpaired Student’s *t*-test, error bars denote SEM, n = 3) (**E**). (**F**) Effects of *TP53I11* transient overexpression on the basal ER Ca^2+^ levels in TuNer-s cells. Typical traces (left). Statistics (right) (****, *p* < 0.0001, unpaired Student’s *t*-test, error bars denote SEM, n = 3). (**G**) Statistics of the luciferase reporter assay results demonstrated the repression of *TP53I11*-3′UTR by 10 ER-Ca^2+^-lowering miRNAs. The assays were conducted in cells co-transfected with the indicated pre-miRNA and one of the following plasmids: *TP53I11*-3′UTR WT-pmiRGLO plasmid, containing the wild-type (WT) *TP53I11*-3′UTR sequence along with luciferase reporters (WT experimental group); *TP53I11*-3′UTR MUT-pmiRGLO plasmid, containing a *TP53I11*-3′UTR sequence with a mutated miRNA-binding site, along with luciferase reporters (mutant experimental group); empty pmiRGLO vector, encoding only luciferase reporters without any *TP53I11*-3′UTR sequence (control group). The firefly luciferase activity (reporter) of each sample was normalized to *Renilla* luciferase activity (expression indicator). A decrease in relative firefly luciferase activity in the presence of ER-Ca^2+^-lowering miRNAs indicates that *TP53I11*-3′UTR contains a target that is modulated by these miRNAs. The dotted line represents the boundary dividing the significant and non-significant areas (***, *p* < 0.001; **, *p* < 0.01; *, *p* < 0.05; ns, no significance, unpaired Student’s *t*-test, error bars denote SEM, n = 3).

Given the lack of a known common regulator for ER Ca^2+^ homeostasis, we sought to identify novel regulators of ER Ca^2+^ levels. We focused on genes unrelated to protein homeostasis that appeared among the top 10 enriched MF and KEGG pathways from a set of 82 DEGs consistently downregulated by all nine miRNAs (Figure 3A, grey; Table 1). This approach led us to select 18 promising candidates for further validation. Additionally, we included hsa-mir-4296, an ER-Ca^2+^-lowering miRNA not analyzed in the initial RNAseq, for comprehensive validation.

We first assessed the impact of the 10 ER-Ca^2+^-lowering miRNAs on the mRNA levels of the 18 candidate genes through RT-qPCR analysis in transfected cells. The results revealed that 3 genes—MARCKS-like 1 gene (*MARCKSL1*), N-myc downstream-regulated gene 1 (*NDRG1*), and tumor protein p53 (TP53)-inducible protein 11 gene (*TP53I11*)—were significantly downregulated compared to the blank control group (Figure 3B). The downregulation of *MARCKSL1*, *NDRG1*, and *TP53I11* highlights their crucial roles and substantial involvement in post-transcriptional regulation mediated by all 10 miRNAs. Additionally, the filamin A gene (*FLNA*) and obscurin-like cytoskeletal adaptor 1 gene (*OBSL1*) were downregulated by 7 out of the 10 miRNAs, suggesting a broader regulatory network and indicating that these miRNAs may modulate gene expression across various pathways.

To further investigate the functional impacts of *MARCKSL1*, *NDRG1*, and *TP53I11*, we performed Ca^2+^ imaging. We designed two shRNAs for each target gene and confirmed their efficacy through RT-qPCR, demonstrating significant knockdown of the respective targets (Figure 3C). Subsequent analysis of the effects of shMARCKSL1#1, shNDRG1#2, and shTP53I11#1 on ER Ca^2+^ levels in HEK TuNer-s cells showed that only cells with shTP53I11#1 knockdown exhibited a significant reduction in basal ER Ca^2+^ levels compared to control cells (Figure 3D,E). This finding indicates that TP53I11 is a critical common target required for the reduction in ER Ca^2+^ levels mediated by all 10 miRNAs. Meanwhile, the predicted secondary structures of the miRNA-target-mRNA duplexes for these nine miRNAs, along with their target site sequences, are shown in Figure S4A–H. Specifically, the target sites of has-miR-4472-2 and has-miR-4516 are almost identical (Figure S4E), while the target sites of has-miR-6763-5p and has-miR-6763-3p are adjacent (Figure S4G). Notably, overexpression of *TP53I11* led to a significant increase in ER Ca^2+^ levels, as evidenced by elevated basal TuNer-s ratios in TuNer-s cells (Figure 3F).

To elucidate the mechanism of miRNA-mediated regulation, we conducted a dual-luciferase reporter assay to determine if these miRNAs directly interact with the 3′UTR of *TP53I11*. HEK293 cells were co-transfected with these miRNAs and a luciferase reporter containing the *TP53I11*-3′UTR or *TP53I11*-3′UTR mutation. Luciferase activity was assessed 48 h post-transfection to assess the impact of miRNA binding on *TP53I11*-3′UTR activity. The results showed that, with the exception of hsa-miR-3183, the other nine miRNAs significantly suppressed luciferase activity driven by the *TP53I11*-3′UTR (Figure 3G). This significant suppression indicates direct binding and regulation of *TP53I11*-3′UTR by these nine miRNAs, while hsa-miR-3183 may target the *TP53I11*-3′UTR indirectly.

Overall, these findings support the notion that TP53I11 is a common target of all 10 ER-Ca^2+^-lowering miRNAs and serves as a key regulator in promoting Ca^2+^ accumulation within the ER.

### 2.4. TP53I11 Regulates ER Ca^2+^ Levels and Cancer Cell Proliferation Under DOX Treatment

*TP53I11* is a recognized TP53-inducible gene with potential tumor-suppressive roles, though its precise mechanisms are not yet fully understood [51]. To explore this, we investigated whether TP53I11 might impact cancer progression by elevating ER Ca^2+^ levels.

**Figure 4:**
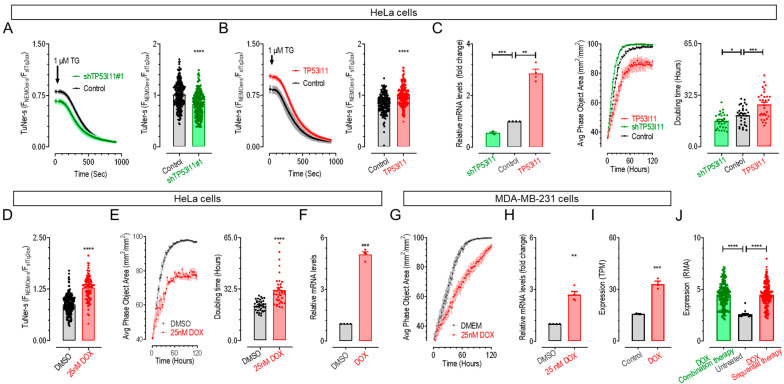
Ca^2+^ imaging and cell proliferation assay revealing TP53I11 boosts DOX’s cell proliferation inhibition by elevating ER Ca^2+^ (**A**,**B**) Effects of *TP53I11* overexpression (**A**) or knockdown (**B**) on resting ER Ca^2+^ levels in HeLa cells. Cells transiently co-expressing TuNer-s together with *TP53I11*, shTP53I11, or an empty vector were imaged. After the recording of baseline, 1 μM TG was applied to see whether ER Ca^2+^ store could be fully depleted in experimental groups. Typical proliferation curves (left). Statistics (right) (****, *p* < 0.0001, unpaired Student’s *t*-test, error bars denote SEM, n = 3). (**C**) Effects of TP53I11 on the proliferation of HeLa cells. Statistics of RT-qPCR results showing the relative mRNA level of *TP53I11* stable overexpression or knockdown HeLa cells (left). Representative proliferation curve (middle). Statistics (right) (***, *p* < 0.001; **, *p* < 0.01; *, *p* < 0.05, unpaired Student’s *t*-test, error bars denote SEM, n = 3). (**D**) A 48 h bath with 25 nM DOX increased basal ER Ca^2+^ levels in HeLa cells transiently expressing TuNer-s. Statistics of basal ER basal Ca^2+^ levels (****, *p* < 0.0001, unpaired Student’s *t*-test, error bars denote SEM, n = 3). (**E**) 25 nM DOX greatly impaired the proliferation of HeLa cells. Typical proliferation curves (left). Statistics (right) (****, *p* < 0.0001, unpaired Student’s *t*-test, error bars denote SEM, n = 3). (**F**) Statistics of RT-qPCR results show that 48 h treatments with 25 nM doxorubicin (DOX), a chemotherapy medication used to treat cancer, upregulated the mRNA levels of *TP53I11* in HeLa cells. The mRNA levels were normalized to GAPDH transcription (***, *p* < 0.001, unpaired Student’s *t*-test, error bars denote SEM, n = 3). (**G**) Cell proliferation assays were used to validate effects of 25 nM DOX. MDA-MB-231 cells were incubated by DOX or DMSO for 48 h. (**H**–**J**) Statistics of RT-qPCR results showing that 48 h treatments with 25 nM DOX upregulated the mRNA levels of *TP53I11* in MDA-MB-231 cells (**H**) (**, *p* < 0.01, unpaired Student’s *t*-test, error bars denote SEM, n = 3). Statistics of *TP53I11* expression levels in MDA-MB-231 cells (GSE222984) (**I**) and tissues from breast cancer patients (GSE137356) (**J**) treated with DOX (****, *p* < 0.0001; ***, *p* < 0.001, unpaired Student’s *t*-test, error bars denote SEM, n = 3).

We used HeLa cells, a well-characterized cancer cell line, to examine how TP53I11 affects ER Ca^2+^ homeostasis using Ca^2+^ imaging. Consistent with what was observed with HEK293 cells (Figure 3D,F), the knockdown of *TP53I11* led to a significant reduction in the TuNer-s ratio in HeLa cells, indicating decreased ER Ca^2+^ levels (Figure 4A). Conversely, overexpression of *TP53I11* led to a notable increase in the TuNer-s ratio, reflecting elevated basal ER Ca^2+^ levels (Figure 4B).

Subsequently, we assessed the impact of TP53I11 on HeLa cell proliferation by comparing the growth of wild-type (WT) cells with those having stable knockdown or overexpression of *TP53I11*. Cells with *TP53I11* knockdown displayed a reduced cell doubling time, while cells with *TP53I11* overexpression exhibited an increased doubling time, indicating accelerated or slowed cell proliferation, respectively (Figure 4C).

These results collectively demonstrate that TP53I11 can elevate basal ER Ca^2+^ levels and inhibit HeLa cell proliferation, suggesting that ER Ca^2+^ modulation could be a potential strategy for cancer intervention strategy. To support this idea, TMCO1 [16], an ER Ca^2+^ leak channel, has been associated with the inhibition of cancer progression through its role in elevating ER Ca^2+^ levels [52]. Additionally, DOX, a broad-spectrum chemotherapeutic agent, has been shown to act as a TP53 agonist and enhance ER Ca^2+^ accumulation [53].

To investigate whether TP53I11 is involved in DOX-induced ER-Ca^2+^ elevation and inhibition of cancer cell proliferation via TP53, we first investigated the effects of 25 nM DOX on ER Ca^2+^ levels and cell growth. Based on a review of the literature and gradient testing, a concentration of 25 nM doxorubicin (DOX) was selected for further experiments [54]. At this concentration, DOX efficiently inhibited cell proliferation while exhibiting minimal cytotoxicity. Higher concentrations induced cell death, which interfered with subsequent proliferation assays. Our results showed a significant increase in the TuNer-s ratio in cells treated with 25 nM DOX compared to the control group, indicating that 25 nM DOX elevates basal ER Ca^2+^ levels (Figure 4D). This finding is consistent with a previous report on MEF cells [53]. Proliferation assays conducted with the IncuCyte Live Cell Analysis System showed a marked increase in cell doubling time for HeLa cells treated with 25 nM DOX compared to DMSO-treated cells, indicating a slowdown in cell proliferation (Figure 4E), which is in line with earlier studies [55,56]. These results confirm that DOX can significantly elevate basal ER Ca^2+^ levels and inhibit cancer cell proliferation.

We next assessed whether DOX could induce upregulation of *TP53I11* with RT-qPCR analysis. The results demonstrated a significant upregulation of *TP53I11* mRNA levels in HeLa cells following 48 h of treatment with 25 nM DOX (Figure 4F). The observed upregulation of *TP53I11* in response to DOX, along with increased ER Ca^2+^ levels and reduced cell proliferation, suggests that TP53I11 plays a crucial role in mediating the effects of DOX on cancer cell proliferation, likely through its capacity to elevate ER Ca^2+^ levels.

Consistent with the aforementioned findings, proliferation assays demonstrated that 25 nM DOX treatment also significantly reduced the proliferation rate of MDA-MB-231 cells, a triple-negative breast cancer (TNBC) cell line, compared to DMSO-treated cells (Figure 4G). This reduction in proliferation was accompanied by an upregulation of *TP53I11* mRNA levels in MDA-MB-231 cells, as evidenced by RT-qPCR (Figure 4H) and RNAseq data from published dataset GSE222984 [54] (Figure 4I).

To further validate the response of TP53I11—given its role as a key regulator of Ca^2+^ homeostasis and a target of ER-Ca^2+^-lowering miRNAs—to DOX, we analyzed transcriptome sequencing data from TNBC patients treated with DOX and cyclophosphamide, another reported activator of TP53 (GSE137356) [57]. Our statistical analysis revealed a significant upregulation of *TP53I11* expression in breast cancer tissues from DOX-treated patients compared to untreated controls, regardless of whether DOX was administered in combination or sequentially with cyclophosphamide (Figure 4J). These findings align with our cell study results and highlight the potential therapeutic value of ER-Ca^2+^ homeostasis in cancer treatment.

## 3. Discussion

Ca^2+^ deregulation has recently emerged as a pivotal element in cancer biology, making Ca^2+^ homeostasis a novel target for cancer therapy [58]. Our study, based on a comprehensive genomic screening, identifies 10 miRNAs that significantly lower basal ER Ca^2+^ levels, a crucial factor for cellular homeostasis. Through RNAseq and Ca^2+^ imaging, we identified that these miRNAs downregulate *TP53I11*, a key tumor suppressor that regulates ER Ca^2+^ levels. Importantly, our function assays confirm that TP53I11 plays a critical role in influencing ER Ca^2+^ levels, thereby affecting cancer cell proliferation. Furthermore, we demonstrated that the chemotherapeutic agent DOX upregulates *TP53I11* expression, leading to enhanced ER Ca^2+^ accumulation. These results not only highlight the central role of TP53I11 in miRNAs-mediated regulation of ER Ca^2+^ homeostasis but also suggest a potential therapeutic strategy targeting ER Ca^2+^ upregulation for cancer intervention.

While our study focuses on ten miRNAs that influence *TP53I11* expression, previous research has highlighted hsa-miR-210-3p, hsa-miR-210-5p, and hsa-miR-645 as other miRNAs targeting TP53I11 [59,60]. These miRNAs were not among the pool of 33 candidate miRNAs found to lower ER Ca^2+^ levels, indicating that their primary targets lie outside the scope of ER Ca^2+^ homeostasis. Nonetheless, the common regulation of TP53I11 by multiple miRNAs emphasizes the intricate epigenetic regulation of this tumor suppressor and its broader implications across various tissues.

Our findings align with previous research linking ER Ca^2+^ homeostasis to cell proliferation. Specifically, we observed that ER Ca^2+^ overload by TP53I11 resulted in slowed HeLa cell proliferation, while reduced basal ER Ca^2+^ levels following *TP53I11* downregulation were associated with enhanced cell proliferation. These observations are supported by studies demonstrating that ER Ca^2+^ overload can induce cell death and reduce tumor growth both in vitro and in vivo [52]. Conversely, reduced ER Ca^2+^ levels could enhance cell proliferation and survival by facilitating SOCE-dependent pathways [61]. While complete depletion of ER Ca^2+^ stores using the Ca^2+^-pump blocker TG leads to cell growth arrest, this is likely due to the ER stress [62]. Our findings suggest that modulating TP53I11 expression and ER Ca^2+^ levels may offer therapeutic avenues for cancer therapy.

Interestingly, our findings reveal that the 10 ER-Ca^2+^-lowering miRNAs target the tumor suppressor TP53I11, potentially positioning these miRNAs as tumor promoters. Evidence supporting this hypothesis includes the observation that high expression of hsa-miR-3687 enhances the migration and invasion of esophageal cancer cells, correlating with poorer patient prognosis [63]. Similarly, elevated hsa-miR-3687 expression is associated with shorter cancer progression time and reduced overall survival in prostate cancer, suggesting a pro-cancer role [64,65]. In bladder cancer, hsa-miR-3687 promotes the growth of bladder cancer cells by downregulating the expression of target gene FOXP subfamily transcription factors and then upregulating the expression of cyclin E2 [66]. These findings support the hypothesis that miRNAs regulating TP53I11 may act as tumor promoters under certain conditions.

However, several miRNAs identified in our study have also been recognized for their anti-cancer roles. For example, hsa-miR-1268b and hsa-miR-4472 are downregulated in breast cancer [67,68], while hsa-miR-4516 deletion is associated with increased cell proliferation [69]. Additionally, hsa-miR-622 has been shown to inhibit cell proliferation, migration, and invasion, suggesting a tumor-suppressive role [70,71,72]. These contrasting effects likely arise from the differential expression levels of miRNAs in different tissues and the diverse range of tissue-specific target genes they regulate [73]. This underscores the complexity of miRNA regulation and suggests that the dual role of miRNAs as both tumor promoters and suppressors warrants further investigation.

Our findings also highlight the role of TP53I11 in modulating cancer cell proliferation. Overexpression of *TP53I11* inhibits cell proliferation, whereas its knockdown accelerates growth. Additionally, DOX treatment significantly increases *TP53I11* expression, suggesting that TP53I11 may contribute to the anti-cancer effects of DOX (Figure 4C–G). These results are consistent with studies indicating that TP53I11 inhibits epithelial–mesenchymal transition and metastasis in breast cancer, with higher TP53I11 levels often associated with a better prognosis [51,74]. Beyond breast cancer, TP53I11 also exhibits tumor-suppressive effects in hepatocellular carcinoma (HCC), where its downregulation correlates with increased cell proliferation and poor prognosis [75,76,77]. Interestingly, *TP53I11* expression was upregulated in cells treated with the anti-tumor agent curcumin, further supporting its role as a tumor suppressor in HCC [78]. However, in gastric cancer, high *TP53I11* expression is associated with poor prognosis [79], indicating that the tumor-suppressive or tumor-promoting role of TP53I11 may be tissue specific. This variability mirrors the divergent roles observed for p53 in cancer cell proliferation [80], which likely arises from its differential regulation in various tissues, interactions with distinct proteins, and specific environmental contexts.

## 4. Materials and Methods

### 4.1. Plasmid Construction

To construct pre-miRNA plasmids, we first designed primers using SnapGene v.1.1.3 software (GSL Biotech LLC, San Diego, CA, USA) and sequences of pre-miRNAs that include miRNA precursors flanked by 100–200 nt, retrieved from miRBase (https://www.mirbase.org/ (accessed on 6 June 2023)) and Ensemble (http://grch37.ensembl.org/index.html (accessed on 6 June 2023)) databases [81,82,83] (Table 2). We next used these primers to PCR-amplify these sequences from a cDNA library generated by the MiniBEST Universal RNA Extraction Kit (Takara, cat. No. 9767, Kusatsu, Japan) and PrimeScript™ RT Master Mix (Takara, cat. No. RR036A, Kusatsu, Japan), and subsequently ligated them with the pCDNA3.1(+)-mScarlet vector [84] linearized with *EcoR I* (Takara, cat. No. 1611, Beijing, China) and *Xho I* (Takara, cat. No. 1635, Beijing, China). A homologous recombination using the Ready-to-Use Seamless Cloning Kit (Sangon Biotech, cat. No. B632219, Beijing, China) was used for the ligation. For those pre-miRNAs that could not be amplified from the cDNA library, corresponding plasmids were constructed by BGI TECH SOLUTIONS (BEIJING LIUHE) Co., Limited (Beijing, China). TP53I11-, MARCKSL1-, and NDRG1-mScarlet were constructed similarly. The PCR reaction was carried out according to the manufacturer’s instructions for Q5^®^ High-Fidelity 2× Master Mix (NEB, cat. No. M0492S, Ipswich, MA, USA) on an ETC811 PCR instrument (Dongsheng Biotech Co., Ltd., ETC811, Guangzhou, China). The reaction mixture (30 µL) consisted of 15 µL of Q5^®^ High-Fidelity 2× Master Mix, 1 µL each of forward and reverse primers (10 µM), and plasmid DNA as the template (50 ng). The PCR amplification conditions were as follows: an initial denaturation at 98 °C for 2 min, followed by 35 cycles of denaturation at 98 °C for 15 s, annealing at 50–68 °C (depending on the primer sequence) for 60 s, and extension at 72 °C for 60 s.

To generate the *TP53I11* knockdown plasmid (shTP53I11), shRNA sequences targeting *TP53I11* obtained from a pool of shRNAs (https://www.sigmaaldrich.cn/CN/zh/semi-configurators/sirna?activeLink=selectAssays (accessed on 12 March 2024)) were synthesized by RUIBIOTECH (RUIBIO BIOTECH Co., Limited, Beijing, China). Subsequently, we inserted these shRNA sequences into the pLKO.1 vector between the *Age I* (NEB, cat. No. R0552, Ipswich, MA, USA) and *EcoR I* restriction sites. A similar approach was employed to generate the shMARCKSL1 and shNDRG1 plasmids (Table 3). To construct plasmids for the dual-luciferase reporter assay, the pmirGLO vector served as the backbone, incorporating both the coding sequences for firefly luciferase, which acted as the reporter, and *Renilla* luciferase, which functioned as the internal reference for expression levels. Subsequently, the 3′UTR of *TP53I11* was synthesized and cloned into the pmirGLO vector by BGI TECH SOLUTIONS (BEIJING LIUHE CO., Limited, Beijing, China) to make the *TP53I11*-3′UTR WT or MUT-pmiRGLO plasmid. All plasmids were confirmed by sequencing.

### 4.2. Cell Culture and Transfection and Generation of Stable Lines

HEK293 cells or TuNer-s cells [43] were cultured in Dulbecco’s modified Eagle medium (DMEM) (Hyclone, Cytiva, cat. No. SH30243.01, 32 Phillip Street, Parramatta, NSW 2150, Australia), containing 10% fetal bovine serum (FBS) (Gibco, cat. No. 16000-044, Grand Island, NY, USA) and 1% Penicillin–Streptomycin Solution (P/S) (Cytiva, cat. No. 15140-122, 32 Phillip Street, Parramatta, NSW 2150, Australia). Cells were grown at 37 °C in a humidified atmosphere containing 5% CO_2_. Notably, TuNer-s cells were cultured in DMEM with 100 µg/mL Geneticin (Sigma-Aldrich, cat. No. 11811-031, Shanghai, China).

For Ca^2+^ imaging, transfection was performed by electroporation using the Bio-Rad Gene Pulser Xcell system in OPTI-MEM medium (Gibco, Thermo Fisher Scientific, cat. No. 31985-070, Waltham, MA, USA). Transfected cells were seeded on round coverslips. Imaging was conducted 24 to 48 h post-transfection.

For RNA-seq, quantitative real-time PCR, and dual-luciferase activity assay, transfection was carried out using Lip2000 Transfection Reagent (Solarbio, cat. No. L7800, Beijing, China). This was conducted according to the manufacturer’s recommended protocols. Transfected cells were seeded on either 6-well (Corning, cat. No. 3516, Corning, NY, USA) or 96-well plates (Corning, cat. No. 3599, Corning, NY, USA). Experiments were carried out 48 h post-transfection.

For generation of multi-clonal stable lines, cells were transfected with corresponding plasmids and selected with 2 µg/mL puromycin for 5–7 days.

### 4.3. Ca^2+^ Imaging

Ca^2+^ imaging experiments were performed using a ZEISS observer Z1 imaging system (ZEISS, Oberkochen, Germany) controlled by SlideBook v.6.0.23 software (Intelligent Imaging Innovations, Inc., Denver, CO, USA) with a rate of 0.5 Hz [44]. A ratiometric genetically encoded Ca^2+^ indicator (GECI) named superfolder mTurquoise2ox (sfTq2^ox^)-NEMOer-s (TuNer-s) was utilized to measure ER Ca^2+^ levels. TuNer-s is composed of NEMOer (Ca^2+^ sensor) and sfTq2^ox^ (a CFP variant that functions as a reference expression level). The following filter settings were used to collect fluorescence of NEMOer-s (F_NEMOer-s_) (500 ± 10 nm _Ex_; 535 ± 15 nm _Em_) and sfTq2^OX^ (F_sfTq2OX_) (438 ± 12 nm _Ex_, 470 ± 12 nm _Em_). Ca^2+^ levels are indicated as F_NEMOer-s_/F_sfTq2OX_ ratio [43].

The Ca^2+^ imaging buffer (pH 7.2) contained 11.5 mM D-Glucose (Xilong Scientific, cat. No. 5996-10-1, Guangzhou, China), 20 mM HEPES (Gamycal, cat. No. A60026-0250, Shanghai, China), 107 mM NaCl (Beijing Chemical Works, cat. No. 7647-14-5, Beijing, China), 7.2 mM KCl (Xilong Scientific, cat. No. 7447-40-7, Guangzhou, China), and 1.2 mM MgCl2 (Xilong Scientific, cat. No. 7791-18-6, Guangzhou, China), and 0.1% Bovine Serum Albumin (BSA) (Sigma-Aldrich, cat. No. 131213, Shanghai, China).

The fluorescence readings from regions of interest (ROI) were exported, analyzed with MATLAB 2014a software (MathWorks, Natick, MA, USA), and plotted using GraphPad Prism 9.51 software (GraphPad Software, San Diego, CA, USA). All experiments were performed at room temperature, and each experiment included a minimum of 30 cells.

### 4.4. RNA-Seq and Bioinformatic Analysis

The test was partitioned into eleven groups: control (HEK293 cells transfected with pCDNA3.1(+)-mScarlet vector) and HEK293 cells transfected with each of 10 pre-miRNAs. Cells were seeded in 6-well plates until they reached a confluence of 50% to 60%. Then, control vectors or pre-miRNAs were transfected into cells using Lip2000 Transfection Reagent. After 48 h transfection, RNA extraction and sequencing were performed by Beijing NovoZone Technology Co. (Beijing, China). Briefly, the RNA samples underwent rigorous quality control by an Agilent 2100 bioanalyzer (Agilent Technologies, Santa Clara, CA, USA). Once the RNA sample is qualified, NEBNext^®^ Ultra™ RNA Library Prep Kit for Illumina^®^ (NEB, cat. No. E7530, Ipswich, MA, USA) was used for library construction. Subsequently, high-throughput sequencing (paired-end 150 nt) was performed on the Illumina NovaSeq 6000 platform (Illumina, San Diego, CA, USA). The reference genome used was hg38. From raw data to clean data, data quality control and filtering were performed using fastp (version 0.23.1). Sequences were aligned to the human reference genome (GRch38/hg38) using HISAT2 (version 2.0.5) [85], and quantification analysis was carried out using featureCounts (version 1.5.0-p3) [86].

For sample correlation analysis, Pearson correlation coefficients were used to quantify the degree of similarity between samples. The cor function and “ggcorrplot” (version 0.1.4.1) facilitated both the correlation analysis and the visualization of results. Three RNA-seq experiments were conducted, with Pearson correlation coefficients clustering applied to each dataset to assess the reliability of the experimental outcomes. The analysis was performed using “ComplexHeatmap” (version 2.16.0), “circlize” (version 2.16.0), and “ggplot2” (version 3.4.2) package [87,88,89].

Differential gene expression analysis was performed using the “DESeq2” package (version 1.40.1) of RStudio 4.3.0 software (Posit, Boston, MA, USA) [90], resulting in log_2_FC, *p*-values (Wald test), and adjusted *p*-values (padjs). The annotation information was sourced from “org.Hs.eg.db” (version 3.17.0). Subsequently, up- and downregulated differentially expressed genes (DEGs) were filtered based on the following criteria: log_2_FC < −0.2 or log_2_FC > 1, and *p*-values < 0.1. Here, log_2_FC < −0.2 indicated downregulated genes, while log_2_FC > 1 indicated upregulated genes. Next, a cluster heatmap of DEGs was generated using the “pheatmap” package (version 1.0.12).

Gene ontology (GO) and Kyoto Encyclopedia of Genes and Genomes (KEGG) enrichment analysis of DEGs identified from RNA-seq were performed using the R package “ClusterProfiler” (version 4.8.1) [91]. Notably, GO enrichment analysis covered 3 categories: molecular function, biological process, and cellular component. The top 10 significant or ion-associated terms (*p*-value < 0.05) were selected and visualized as bubble plots, arranged in descending order of GeneRatio.

For the transcriptomic analysis of expression levels in datasets GSE222984 and GSE137356, gene expression data were initially retrieved from the GEO database (https://www.ncbi.nlm.nih.gov/geo/ (accessed on 18 June 2024)) [92,93]. Following data acquisition, computational analysis of the RNA-seq data was performed using Galaxy v.22.05.1 (https://usegalaxy.org (accessed on 18 June 2024)) [94], with gene counts scaled and normalized to TPM (Transcripts Per Kilobase Million) and RMA (Robust Multi-array Average) units, which were used for further analysis.

### 4.5. Quantitative Real-Time PCR (RT-qPCR)

The pre-miRNAs (10 μg) or empty vector (1 μg) were transfected into HEK293 cells using Lip2000 Transfection Reagent when the cells reached approximately 60% confluence in 6-well plates. A total of 48 h post-transfection, total RNA was isolated using a miniBEST Universal RNA Extraction Kit (Takara, cat. No. 9767, Beijing, China). First-strand cDNA was synthesized from 1 mg of total RNA using PrimeScript™ RT Master Mix (Takara, cat. No. RR036A, Kusatsu, Japan). Primers for human glyceraldehyde-3-phosphate dehydrogenase (*GAPDH*) and the target genes were designed using the Primer3Plus-Pick Primers (https://www.primer3plus.com/ (accessed on 14 November 2023)) website [95] (Table 4). The quantitative real-time PCR reaction system and conditions were established following the instructions for 2× RealStar Green Power Mixture (with ROX II) (GenStar, cat. No. A314-05, Beijing, China) and performed on the ABI Quant Studio™ 6 Flex (ABI, Carlsbad, CA, USA). The reaction mixture (10 µL) contained 5 µL of 2× RealStar Green Power Mixture (with ROX II) and 0.25 µL each of forward and reverse primers (10 pM). The RT-qPCR program was set at 95 °C for 10 min, followed by 40 cycles of 95 °C for 15 s and 60 °C for 60 s. Relative gene expression was quantified by the 2^−ΔΔCt^ method [96], and fold change in gene expression under stress conditions was calculated relative to control samples. The gene expression was normalized to the expression of *GAPDH* as the reference genes.

### 4.6. Dual-Luciferase Reporter Assay

RNAhybrid (https://bibiserv.cebitec.uni-bielefeld.de/rnahybrid/ (accessed on 31 October 2024)) [95] was used to predict potential miRNA target sites within the viral sequence [97]. One key criterion was applied: minimum free energy (mfe) ≤ −25 kcal/mol. Based on the predicted sites, miRNA-binding mutation sites were designed and synthesized by RiboBio Co., Ltd. (Beijing, China), followed by the construction of a pmiRGLO plasmid containing the mutation sites (*TP53I11*-3′UTR MUT-pmiRGLO). To examine whether the *TP53I11*-3′UTR functions as a target of ER-Ca^2+^-lowering miRNAs in vitro, dual-luciferase assays were performed using a pmiRGLO Dual-Luciferase miRNA Target Expression Vector (Promega, cat. No. E1330, Fitchburg, WI, USA), containing both the coding sequences of firefly luciferase (reporter) and *Renilla* luciferase (internal control). A total of 24 h after being plated in 96-well plates, HEK293 cells were transfected with either pCDNA3.1(+)-mScarlet vector (blank control), pre-miRNAs together with an empty pmiRGLO vector (control), or *TP53I11*-3′UTR WT or MUT-pmiRGLO with Lip2000 Transfection Reagent following the abovementioned protocols (5 μg DNA each). A total of 48 h post-transfection, the cells were rinsed with PBS and harvested according to the manufacturer’s protocol of the duo-lite luciferase assay system (Vazyme, cat. No. DD1205-01, Nanjing, China). Luciferase activities were measured on the FlexStation3 (Molecular Devices, San Jose, CA, USA). Luminescence from firefly luciferase activity was measured at 540–600 nm, while that from *Renilla* luciferase activity was measured at 460–540 nm. The firefly *Renilla* luciferase activity was normalized against *Renilla*. The resulting normalized firefly luciferase activity (firefly luciferase activity/*Renilla* luciferase activity) for each construct was then compared with that of the control group.

### 4.7. Cell Proliferation Assay

To evaluate the role of DOX (Beijing PolySciences, cat. no. D95236, Beijing, China) in the proliferation of tumor cells, approximately 5000–10,000 HeLa, MDA-MB-231 cells or multi-clonal stable cells were seeded into 96-well flat-bottomed plates. These cells were incubated for 48 h either in the presence of 25 nM DOX, an anti-tumor drug, or 0.01% DMSO [54]. The real-time proliferation of those seeded cells was imaged by the IncuCyte Live Cell Analysis System (Essen BioScience, Inc., Ann Arbor, MI, USA) every 2 h. Four days later, the confluence (%) readings per pore cell were analyzed with IncuCyte S3 2018B software (Essen BioScience, Inc., Ann Arbor, MI, USA) and plotted using GraphPad Prism 9.5.1 software.

### 4.8. Statistics

Statistical analysis and visualization were performed using GraphPad Prism 9.51 software. In this study, a comparison between the two groups was made using an unpaired, two-tailed Student’s *t*-test. Significant differences are indicated in the figure legends. * *p* < 0.05, ** *p* < 0.01, *** *p* < 0.001, **** *p* < 0.0001, ns indicates not significant. Unless otherwise specified, all data were expressed as mean ± SEM. All experiments were performed in triplicate.

## 5. Conclusions

In this study, we identified 10 miRNAs from 33 candidates obtained through previous CRISPR screening that lower basal ER Ca^2+^ levels by targeting the tumor suppressor TP53I11. Overexpression of these 10 miRNAs significantly reduced basal ER Ca^2+^ levels, with TP53I11 as a common target, and functional assays confirmed that TP53I11 regulates ER Ca^2+^ homeostasis. Additionally, TP53I11 modulates ER Ca^2+^ levels and cancer cell growth during chemotherapy, particularly with doxorubicin (DOX), which upregulates TP53I11 and enhances ER Ca^2+^ accumulation. These findings highlight the crucial role of TP53I11 in ER Ca^2+^ regulation and suggest that the miRNA-mediated modulation of TP53I11 could offer a promising therapeutic strategy in cancer treatment. Future studies should explore the miRNA-TP53I11 regulatory network to develop more effective cancer therapies.

## Figures and Tables

**Table 1:** 82 commonly downregulated DEGs.

Gene Name
*NDUFS6*/*RTL8A*/*TNIK*/*NRP1*/*C20orf27*/*FASN*/*KIAA1522*/*TIMP3*/*PDCD4*/*CTDSP2*/*PXDN*/*CLPTM1*/*ASF1B*/*PODXL2*/ *CAPN1*/*JAG1*/*GLG1*/*MYO10*/*RCC2*/*LLGL1*/*HNRNPA0*/*SOX12*/*AKR7A2*/*SPEN*/*TRIM56*/*KDELR1*/*NCSTN*/*DHTKD1*/*ACLY*/ *SUMF2*/*RTL8C*/*CDK16*/*CRAT*/*TGFB1*/*DAG1*/*NUDT16*/*BSG*/*ISYNA1*/*NUP210*/*BIN1*/*PPP1CA*/*PARP1*/*SMOC2*/*NDE1*/ *RTN4*/*OBSL1*/*DPP3*/*RBCK1*/*PLXNA2*/*PSAP*/*LAMB1*/*LRP1*/*NDRG1*/*SDC1*/*ENDOD1*/*RPA1*/*ABCC5*/*JARID2*/*DBF4B*/*ALX4*/ *PRSS23*/*TRIOBP*/*CD81*/*ATP2B4*/*TP53I11*/*TIMELESS*/*SDC3*/*KRT19*/*MARCKSL1*/*ABCA2*/*TKFC*/*MBOAT7*/*COL6A1*/ *DDR1*/*UHRF1*/*MAGED1*/*FLNA*/*REEP2*/*APP*/*TMTC2*/*NRSN2*/*KHNYN*

**Table 2:** Primers of pCDNA3.1(+)–pre-miRNA–mScarlet plasmid constructions.

miRNAs	Primer Name	Sequence (5′→3′)
hsa-mir-622	F-mir-622	ctagtccagtgtggtggaattcTAGCAGGGAGACAGAGATCGAGG
	R-mir-622	gccgccacctctagagatatcACTCCTGGGCCTGGCGC
hsa-mir-1268b	F-mir-1268b	tagtccagtgtggtggaattcTTTCTCGCGCCTTCC
	R-mir-1268b	gccgccacctctagagatatcGCTCCGAGCCCCGCC
hsa-mir-3183	F-mir-3183	tccagtgtggtggaattcCGTTTCCTGGGGCTCCC
	R-mir-3183	gccgccacctctagagatatcCTTGGCTGCAGGACACCTT
hsa-mir-3687	F-mir-3687	tagtccagtgtggtggaattcTTTCTCGCGCCTTCC
	R-mir-3687	gccgccacctctagagatatcGCTCCGAGCCCCGCC
hsa-mir-4296	F-mir-4296	tagtccagtgtggtggaattcCCTGTAGAGACAGGC
	R-mir-4296	aagccgccacctctagagatatcCGAGACTTGGGAGTG
hsa-mir-4472-2	F-mir-4472-2	tccagtgtggtggaattcTTGGGAGGCTGAGGCAGG
	R-mir-4472-2	gccgccacctctagagatatcTTTATCAGCAAGGTCTTTATCACCTGT
hsa-mir-4516	F-mir-4516	tagtccagtgtggtggaattcTAAAAATACAAAATTAGCCAGG
	R-mir-4516	gccgccacctctagagatatcTCACGCAGGTGCCTC
hsa-mir-5708	F-mir-5708	tccagtgtggtggaattcCCATTGCAGCCTCAACCTCC
	R-mir-5708	gccgccacctctagagatatcGTGAGATCTTGGTTCACTGCAACC
hsa-mir-6763	F-mir-6763	tagtccagtgtggtggaattcTTTGGGGGCCTGGGC
	R-mir-6763	gccgccacctctagagatatcCTGCTCCTCCTTGCC
hsa-mir-7851	F-mir-7851	tagtccagtgtggtggaattcATTCCCAACTTAACG
	R-mir-7851	gccgccacctctagagatatcCCAACTTGCCTTTTT

**Table 3:** shRNA sequences of *TP53I11*, *MARCKSL1,* and *NDRG1.*

Reference Sequence ID	Gene Name	shRNA Name	Sequence (5′→3′)
NM_001258320.2	*TP53I11*	shTP53I11#1	GATCATGTGGAACGCTCTCTA
		shTP53I11#2	GCTCACCGAAGCTTGCTATTT
NM_023009.7	*MARCKSL1*	shMARCKSL1#1	CTTCAAGAGAAATCGGAAGGA
		shMARCKSL1#2	CTTTCAAATTGAGCGGCCTGT
NM_001135242.2	*NDRG1*	shNDRG1#1	CCTGGAGTCCTTCAACAGTTT
		shNDRG1#2	GCACATTGTGAATGACATGAA

**Table 4:** Primers for RT-qPCR of 18 candidate genes.

Reference Sequence ID	Gene Name	Primer Name	Sequence (5′→3′)
NM_000484.4	*APP*	APP-QF	CGAGGACGATGAGGATGGTG
		APP-QR	CACACCTCTCGAACCACCTC
NM_001728.4	*BSG*	BSG-QF	CTCCCAGAGTGAAGGCTGTG
		BSG-QR	CTTGTACCAGGCCCAGTCAG
NM_000755.5	*CRAT*	CRAT-QF	CCCTCACTGCGGATCAGATC
		CRAT-QR	GGTGGTTGGAGGTGAGGATG
NM_001165928.4	*DAG1*	DAG1-QF	GAACCAGGACACCATGGGAG
		DAG1-QR	GTGGGACATAGGGAGGAGGT
NM_004104.5	*FASN*	FASN-QF	AAGGAGGGTGTGTTTGCCAA
		FASN-QR	CACCTTCTTGAGCTCCTGCA
NM_001110556.2	*FLNA*	FLNA-QF	CTGCTCGGTCGAGTACATCC
		FLNA-QR	CACATCATGCACAGGGACCT
NM_023009.7	*MARCKSL1*	MARCKSL1-QF	CACTGCTCAGGAAGGGAAGG
		MARCKSL1-QR	GCCCTGCCTCTTCTTCTGAG
NM_012334.3	*MYO10*	MYO10-QF	GCGACTACGACTACGACCAG
		MYO10-QR	GTTGTAGGTCCCCACAGAGC
NM_015331.3	*NCSTN*	NCSTN-QF	CCAGGGCCCTTTGCATTCTA
		NCSTN-QR	CGGATATCTTTCCAGCGGCT
NM_001135242.2	*NDRG1*	NDRG1-QF	TGGTTCCAGCGTCACTTCTC
		NDRG1-QR	GAGTTGGGGGTGATGTCCAG
NM_003873.7	*NRP1*	NRP1-QF	GCCAGAGGAGTACGATCAGC
		NRP1-QR	TCATCCACAGCAATCCCACC
NM_015311.3	*OBSL1*	OBSL1-QF	AGGACAGTGGCGAGTTTGAG
		OBSL1-QR	CGGAAGTTATGGCATGCACG
NM_001008709.2	*PPP1CA*	PPP1CA-QF	CCGTGGCGTCTCTTTTACCT
		PPP1CA-QR	AACTCGCCACAGTAGTTGGG
NM_020532.5	*RTN4*	RTN4-QF	ATGAAGGCCACCCATTCAGG
		RTN4-QR	AAGAAGAGGCGCCTGAGTTC
NM_000660.7	*TGFB1*	TGFB1-QF	GCGTGCTAATGGTGGAAACC
		TGFB1-QR	GAGCAACACGGGTTCAGGTA
NM_152588.3	*TMTC2*	TMTC2-QF	GCAGCAGTCACTGGTCTCTT
		TMTC2-QR	CCGTGTGAATGGGGTGAGAA
NM_001258320.2	*TP53I11*	TP53I11-QF	ATCGCTCCAAGATCAGCCAG
		TP53I11-QR	GAGCACAGCAGAGACGAACT
NM_001039141.3	*TRIOBP*	TRIOBP-QF	TCCACACCAAGGATGCTGTC
		TRIOBP-QR	TTCTGGGTGCTGTAGCTGTG
NM_002046.7	*GAPDH*	GAPDH-QF	AACTGCTTAGCACCCCTGGC
		GAPDH-QR	ATGACCTTGCCCACAGCCTT

## Data Availability

The data that support the findings of this study are available upon reasonable request. The transcriptome data were deposited in the NCBI SRA database with accession numbers SRR31285392, SRR31285394, SRR31285415, SRR31285416, SRR31285404, SRR31285417, SRR31285409, SRR31285395, SRR31285401, SRR31285410, SRR31285396, SRR31285399, SRR31285400, SRR31285407, SRR31285405, SRR31285402, SRR31285403, SRR31285413, SRR31285408, SRR31285397, SRR31285398, SRR31285406, SRR31285414, SRR31285393, SRR31285412, SRR31285411 under the BioProject accession number PRJNA1182663.

## Supplementary Materials

The following supporting information can be downloaded at: https://www.mdpi.com/article/10.3390/ijms26010031/s1.
