# Supplementary material for: TP53I11 Functions Downstream of Multiple MicroRNAs to Increase ER Calcium Levels and Inhibits Cancer Cell Proliferation
Source: Int J Mol Sci. 2024 Dec 24;26(1):31. doi: 10.3390/ijms26010031 (PMC11719883; doi:10.3390/ijms26010031)
Supplement: Supplementary file 1 [file ijms-26-00031-s001.zip › ijms-3389716-supplementary.pdf]

Figure S1

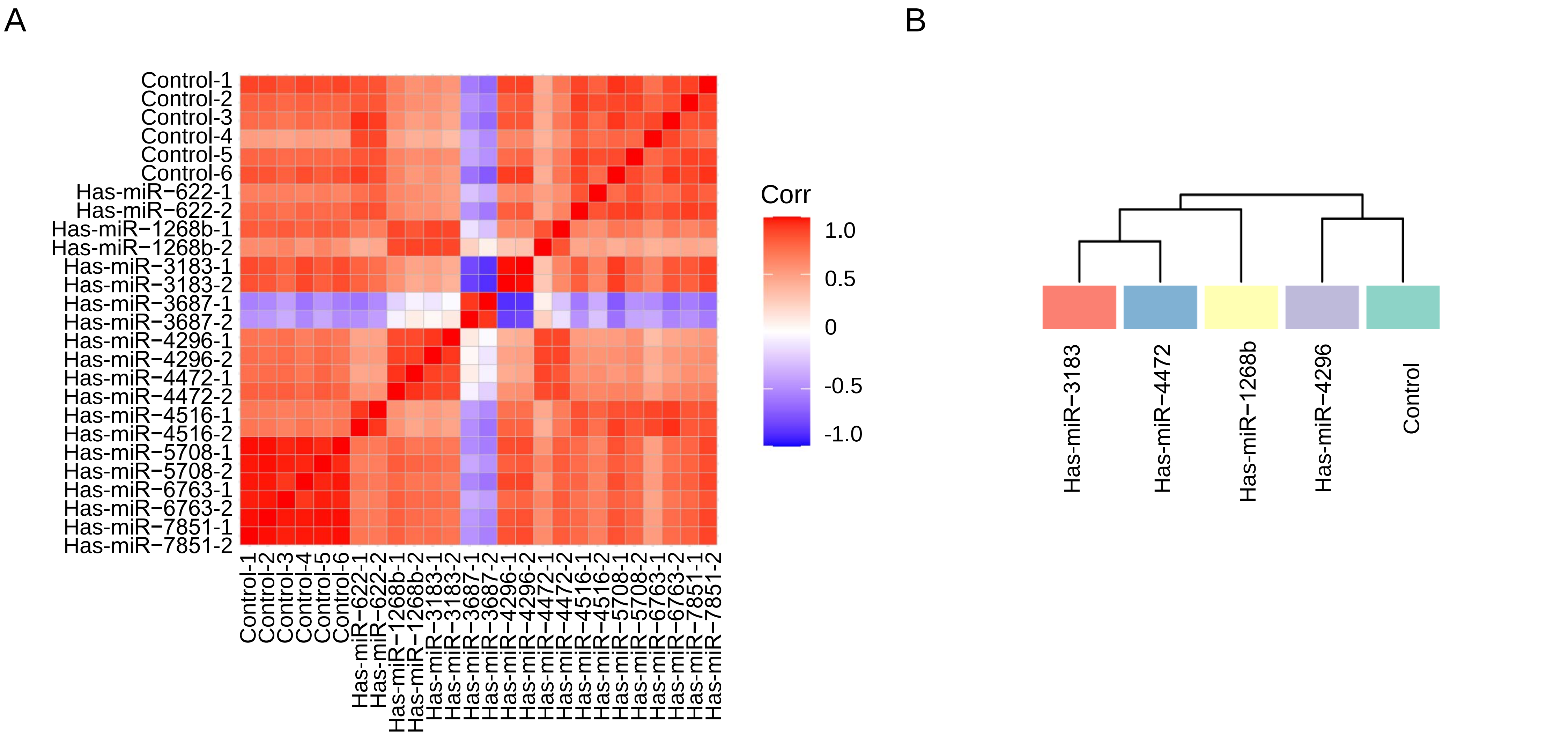
A
B
Control-1
Control-2
Control-3
Control-4
Control-5
Control-6
Has-miR−622-1
Has-miR−622-2
Has-miR−1268b-1
Has-miR−1268b-2
Has-miR−3183-1
Has-miR−3183-2
Has-miR−3687-1
Has-miR−3687-2
Has-miR−4296-1
Has-miR−4296-2
Has-miR−4472-1
Has-miR−4472-2
Has-miR−4516-1
Has-miR−4516-2
Has-miR−5708-1
Has-miR−5708-2
Has-miR−6763-1
Has-miR−6763-2
Has-miR−7851-1
Has-miR−7851-2
Corr
1.0
0.5
0
-0.5
-1.0
Has-miR−3183
Has-miR−4472
Has-miR−1268b
Has-miR−4296
Control

Figure S2

A

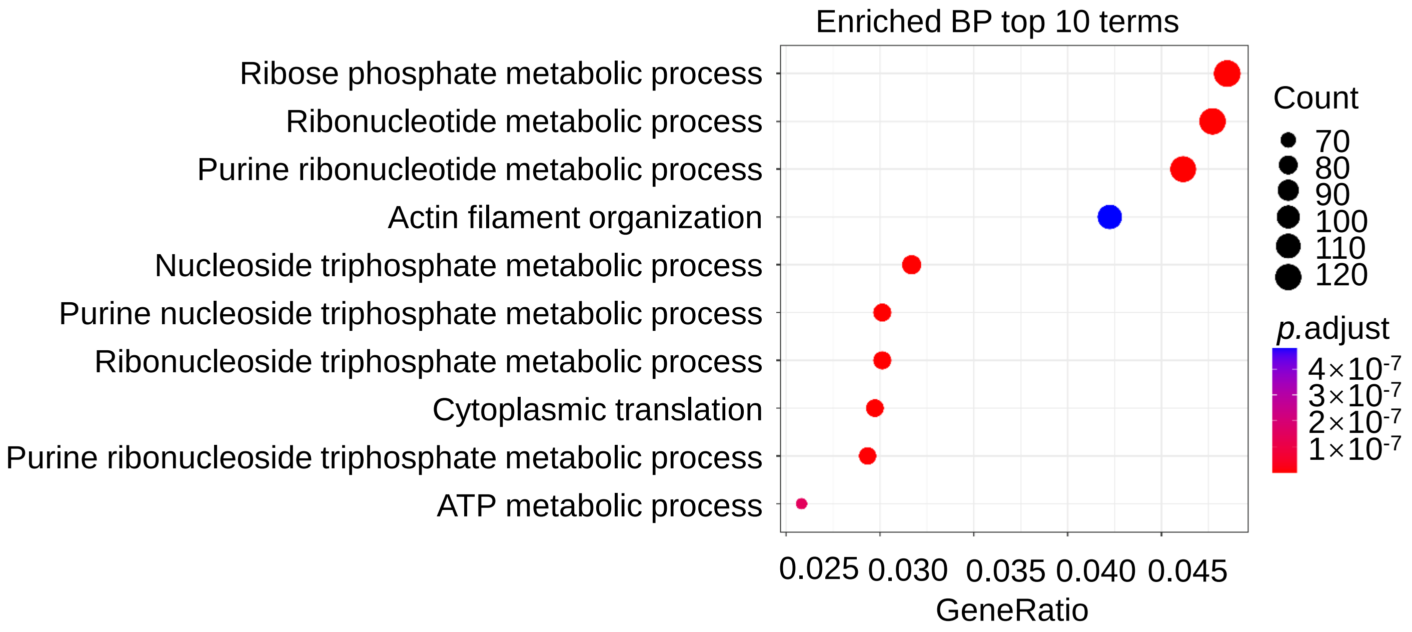
Enriched BP top 10 terms
Ribose phosphate metabolic process
Ribonucleotide metabolic process
Purine ribonucleotide metabolic process
Actin filament organization
Nucleoside triphosphate metabolic process
Purine nucleoside triphosphate metabolic process
Ribonucleoside triphosphate metabolic process
Cytoplasmic translation
Purine ribonucleoside triphosphate metabolic process
ATP metabolic process
0.025 0.030 0.035 0.040 0.045
GeneRatio
Count
70
80
90
100
110
120
p.adjust
4×10-7
3×10-7
2×10-7
1×10-7

B

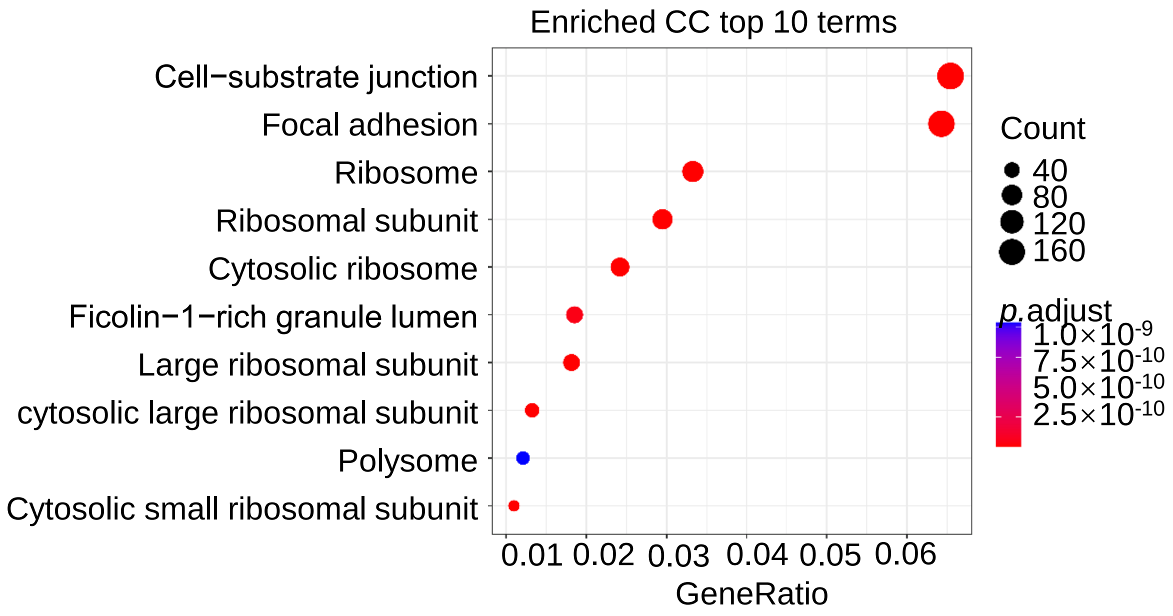
Enriched CC top 10 terms
Cell−substrate junction
Focal adhesion
Ribosome
Ribosomal subunit
Cytosolic ribosome
Ficolin−1−rich granule lumen
Large ribosomal subunit
cytosolic large ribosomal subunit
Polysome
Cytosolic small ribosomal subunit
0.01 0.02 0.03 0.04 0.05 0.06
GeneRatio
Count
40
80
120
160
p.adjust
1.0×10-9
7.5×10-10
5.0×10-10
2.5×10-10

Figure S3

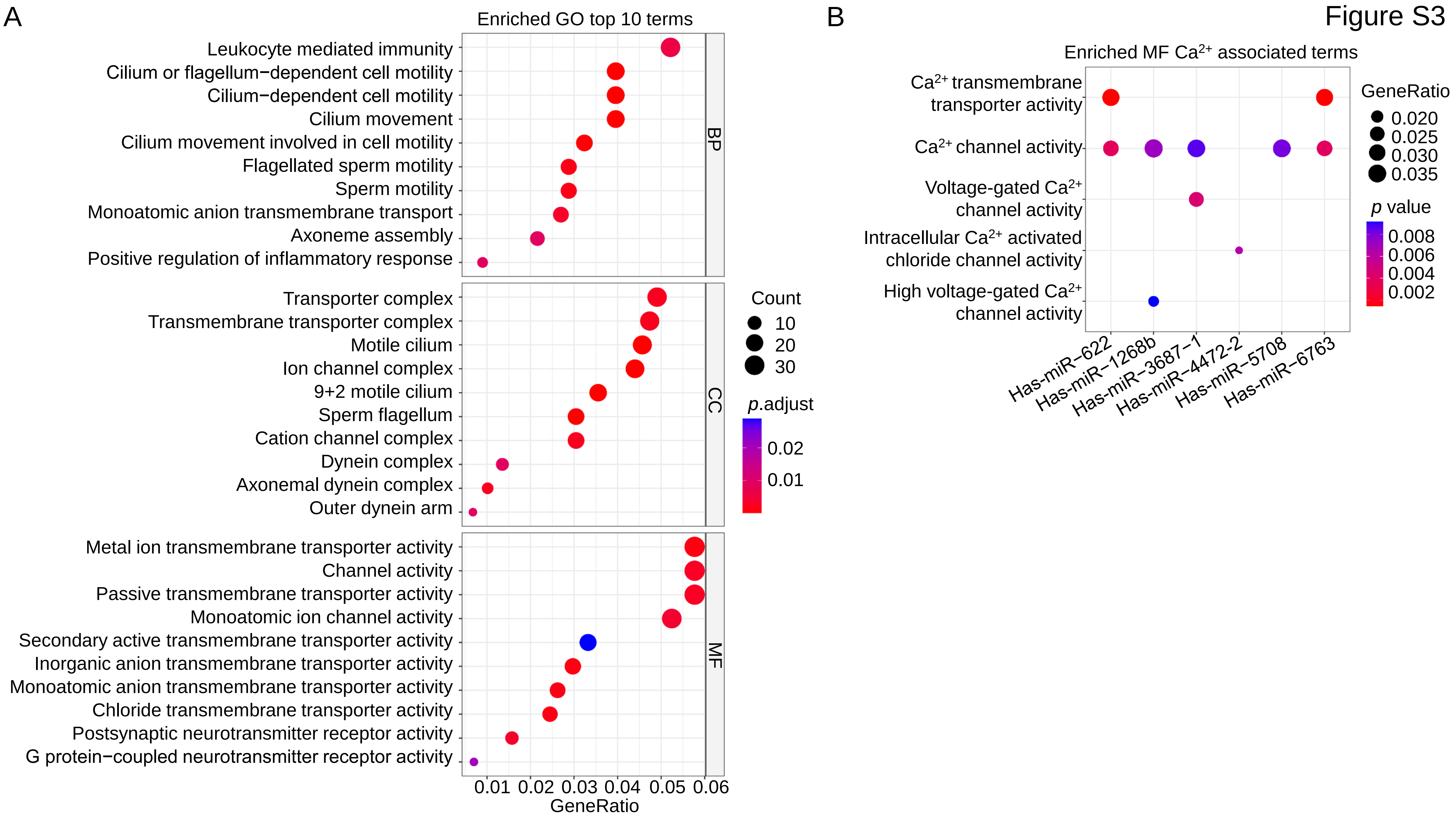

A
Enriched GO top 10 terms
Leukocyte mediated immunity
Cilium or flagellum−dependent cell motility
Cilium−dependent cell motility
Cilium movement
Cilium movement involved in cell motility
Flagellated sperm motility
Sperm motility
Monoatomic anion transmembrane transport
Axoneme assembly
Positive regulation of inflammatory response
BP
Transporter complex
Transmembrane transporter complex
Motile cilium
Ion channel complex
9+2 motile cilium
Sperm flagellum
Cation channel complex
Dynein complex
Axonemal dynein complex
Outer dynein arm
CC
Metal ion transmembrane transporter activity
Channel activity
Passive transmembrane transporter activity
Monoatomic ion channel activity
Secondary active transmembrane transporter activity
Inorganic anion transmembrane transporter activity
Monoatomic anion transmembrane transporter activity
Chloride transmembrane transporter activity
Postsynaptic neurotransmitter receptor activity
G protein−coupled neurotransmitter receptor activity
MF
0.01 0.02 0.03 0.04 0.05 0.06
GeneRatio
Count
10
20
30
p.adjust
0.02
0.01
B
Enriched MF Ca2+ associated terms
Ca2+ transmembrane transporter activity
Ca2+ channel activity
Voltage-gated Ca2+ channel activity
Intracellular Ca2+ activated chloride channel activity
High voltage-gated Ca2+ channel activity
Has-miR−622
Has-miR−1268b
Has-miR−3687−1
Has-miR−4472-2
Has-miR−5708
Has-miR−6763
GeneRatio
0.020
0.025
0.030
0.035
p value
0.008
0.006
0.004
0.002

Figure S4

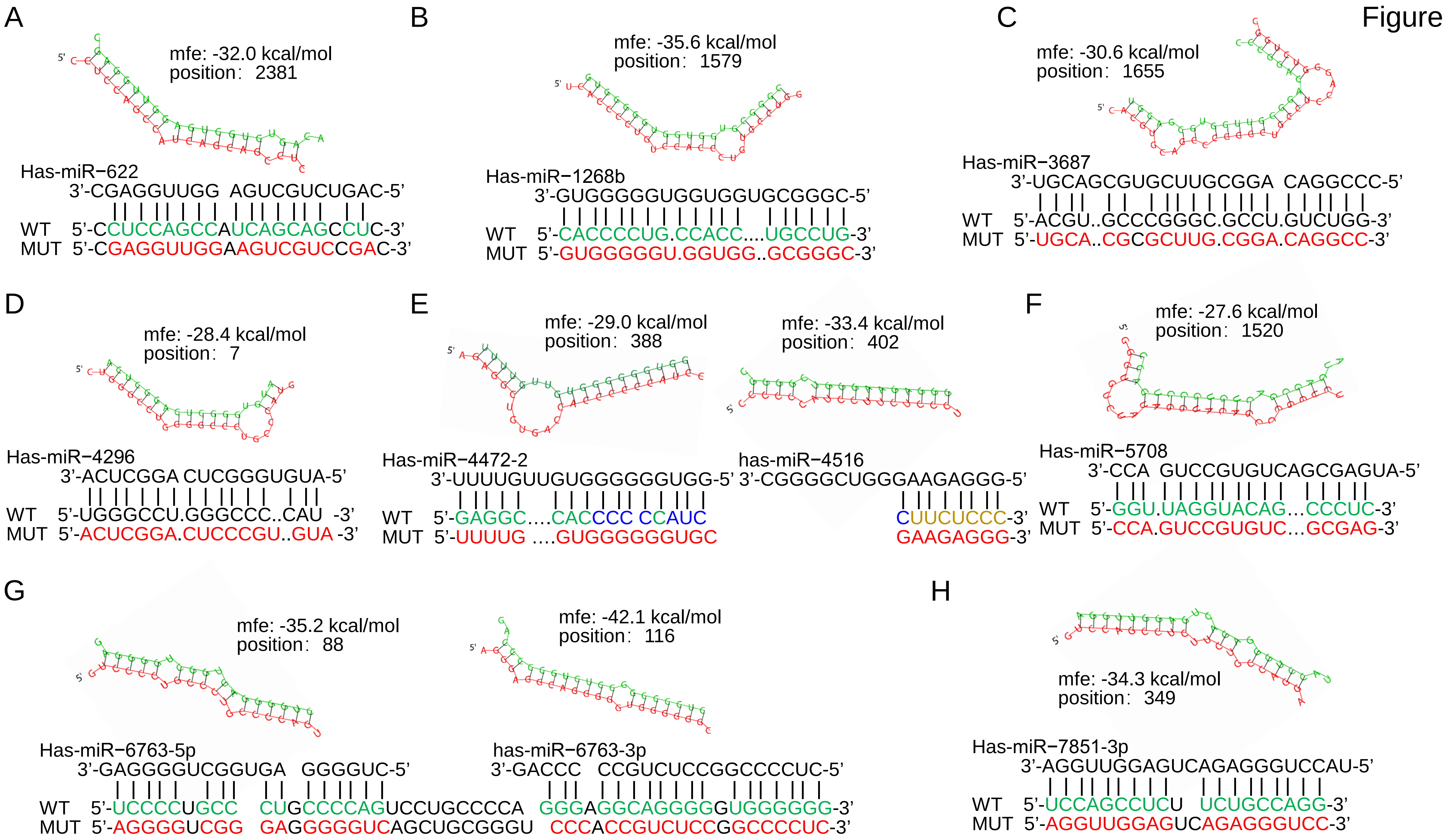
A
mfe: -32.0 kcal/mol
position: 2381
Has-miR−622
3'-CGAGGUUGG AGUCGUCUGAC-5'
WT 5'-CCUCCAGCCAUCAGCAGCCUC-3'
MUT 5'-CGAGGUUGGAAGUCGUCCGAC-3'
B
mfe: -35.6 kcal/mol
position: 1579
Has-miR−1268b
3'-GUGGGGGUGGUGGUGCGGGC-5'
WT 5'-CACCCCUG.CCACC....UGCCUG-3'
MUT 5'-GUGGGGGU.GGUGG..GCGGGC-3'
C
mfe: -30.6 kcal/mol
position: 1655
Has-miR−3687
3'-UGCAGCGUGCUUGCGGA CAGGCCC-5'
WT 5'-ACGU..GCCCGGGC.GCCU.GUCUGG-3'
MUT 5'-UGCA..CGCGCUUG.CGGA.CAGGCC-3'
D
mfe: -28.4 kcal/mol
position: 7
Has-miR−4296
3'-ACUCGGA CUCGGGUGUA-5'
WT 5'-UGGGCCU.GGGCCC..CAU -3'
MUT 5'-ACUCGGA.CUCCCGU..GUA -3'
E
mfe: -29.0 kcal/mol
position: 388
Has-miR−4472-2
3'-UUUUGUUGUGGGGGGUGG-5'
WT 5'-GAGGC....CACCCC CCAUC
MUT 5'-UUUUG ....GUGGGGGGUGC
mfe: -33.4 kcal/mol
position: 402
has-miR−4516
3'-CGGGGCUGGGAAGAGGG-5'
CUUCUCCC-3'
GAAGAGGG-3'
F
mfe: -27.6 kcal/mol
position: 1520
Has-miR−5708
3'-CCA GUCCGUGUCAGCGAGUA-5'
WT 5'-GGU.UAGGUACAG...CCCUC-3'
MUT 5'-CCA.GUCCGUGUC...GCGAG-3'
G
mfe: -35.2 kcal/mol
position: 88
Has-miR−6763-5p
3'-GAGGGGUCGGUGA GGGGUC-5'
WT 5'-UCCCCUGCC CUGCCCCAGUCCUGCCCCA
MUT 5'-AGGGGUCGG GAGGGGGUCAGCUGCGGGU
mfe: -42.1 kcal/mol
position: 116
has-miR−6763-3p
3'-GACCC CCGUCUCCGGCCCCUC-5'
GGGAGGCAGGGGGUGGGGGG-3'
CCCACCGUCUCCGGCCCCUC-3'
H
mfe: -34.3 kcal/mol
position: 349
Has-miR−7851-3p
3'-AGGUUGGAGUCAGAGGGUCCAU-5'
WT 5'-UCCAGCCUCU UCUGCCAGG-3'
MUT 5'-AGGUUGGAGUCAGAGGGUCC-3'

Figure S1.
(A) Heatmap of correlation matrices for ER-Ca^{2+}-lowering miRNAs. The x-axis and y-axis denote RNA-sequencing samples, with the color intensity of the squares representing Pearson correlation coefficients (R). Deeper red indicates stronger correlations.
(B) Dendrogram of gene clustering. Different colors denote distinct groups, with closely related groups clustering together on the same branch.
Figure S2.
(A-B) BP (A) and CC (B) enrichment analysis for down-regulated DEGs from each miRNAs transfected HEK293 cells. Dot plots showing the top 10 terms.
Figure S3.
(A-B) GO enrichment analysis for up-regulated DEGs from each miRNAs transfected HEK293 cells. Dot plots showing the top 10 terms (A), as well as Ca^{2+} associated MF terms (B).
Figure S4.
(A-H) Structures, binding sites and mutation site of miRNAs in TP53I11-3'UTR with mfe ≤ -25 kcal/mol.
